# Effect of Calcium Hydroxide on the Push-out Bond Strength of Endodontic Biomaterials in Simulated Furcation Perforations

**DOI:** 10.7508/iej.2016.02.003

**Published:** 2016-03-20

**Authors:** Negin Ghasemi, Mohammad Forough Reyhani, Amin Salem Milani, Hadi Mokhtari, Faezeh Khoshmanzar

**Affiliations:** a *Dental and Periodontal Research Center, Department of Endodontics, Dental School, Tabriz University of Medical Sciences, Tabriz, Iran; *; b * Department of Endodontics, Dental School, Tabriz University of Medical Sciences, Tabriz, Iran;*; c * Dental School, Tabriz University of Medical Sciences, Tabriz, Iran*

**Keywords:** Calcium-Enriched Mixture, Calcium Hydroxide, Furcation Perforation, Mineral Trioxide Aggregate, Push-Out

## Abstract

**Introduction:**

The aim of this *in vitro* study was to evaluate the effect of calcium hydroxide (CH) on push-out bond strength of white mineral trioxide aggregate (WMTA) and calcium-enriched mixture (CEM) cement in simulated furcation perforations.

**Methods and Materials:**

Furcation perforations, measuring 1.3 mm in diameter and 2 mm in height, were created in 80 human mandibular first molars. The teeth were then divided into 4 groups (*n*=20). In groups 1 and 3 CH was placed in the perforation for one week, before placement of WMTA and CEM. In groups 2 and 4 perforations were repaired without placement of CH. In groups 1 and 2 the perforation sites were repaired with WMTA and CEM cement was used in groups 3 and 4. After 7 days, the push-out test was carried out using a universal testing machine. Data were analyzed with two-way ANOVA. The level of significance was set at 0.05.

**Results:**

The maximum and minimum bond strength values were recorded in the WMTA/CH (13.08±1.8 MPa) and CEM cement groups (8.03±0.98 MPa), respectively. There were significant differences in resistance to dislodgement between the WMTA/CH and other groups (*P*<0.05).

**Conclusion:**

Placement of CH before placement of WMTA in furcation perforation improves the push-out bond strength of this material.

## Introduction

Iatrogenic perforation is one of the challenges for endodontists during root canal treatment [1, 2]. These procedural errors are among the most important reasons for the failure of endodontic treatments and have different prognoses based on the location and size of perforation. Furcation perforations have a higher risk for loss of periodontal attachments and bone loss due to their proximity to the gingival sulcus and therefore require more attention [3]. 

A large number of studies have focused on different repair materials used to close and seal the aberrant communication iatrogenically created between the periodontium and the root canal space. White mineral trioxide aggregate (WMTA) and calcium-enriched mixture (CEM) cement have been successfully used as endodontic biomaterials to seal off the perforation site [4-9]. These two materials are composed of different chemical compositions of calcium and studies have shown favorable outcomes with them [1, 4, 5, 8, 10-12]. 

Ideal perforation repair materials should be well tolerated by periodontal tissues, manipulated easily, exhibit dimensional stability and radiopacity, set in the presence of blood and moisture, provide proper seal and have proper marginal adaptation with the walls of the perforation [8, 10]. On the other hand, the bond strength of these materials with the walls of the perforation site is of utmost importance because they should resist dislodgment due to the application of occlusal forces and the forces resulting from packing of restorative materials to be able to maintain a proper seal in the perforated area. Based on the results of various studies, the push-out test is a reliable technique to evaluate the bond strength [2, 13-17]. 

The push-out bond strength of the two biomaterials mentioned above has been evaluated in different studies, which have focused on various factors affecting the bond strength. Some of the factors evaluated in this respect are the different techniques used to mix the materials [18], the acidic [10] or alkaline environment [19], presence of phosphate-buffered saline [20], blood contamination [21] and the presence or absence of the smear layer [8]. Changes in the pH of host tissues might affect the physical and chemical properties of the materials. Based on the results of previous studies, these changes include a decrease in the material hardness, loss of the sealing ability and a decrease in compressive strength [22, 23].

Given changes in the pH of the environment, one of the important steps in perforation management is to place calcium hydroxide (CH) in the perforation area, which is warranted considering the possibility of harbored bacteria being present in that area and the presence of an acidic environment (inflammation), especially in old perforations [12, 24, 25]. In a study, placement of CH in the perforation area resulted in an improvement in the marginal adaptation of the WMTA apical plug [25]. The aim of the present *in vitro *study was to evaluate the effect of CH on the push-out bond strength of WMTA and CEM cement used for repairing of the furcation perforations.

## Materials and Methods

***Selection of teeth and preparation of samples***

Eighty mandibular first molars were selected. The inclusion criteria consisted of no root fusions, no morphological or size anomalies, no carious lesions in the furcation area and no previous root canal treatment. After removing the soft tissues, the teeth were stored in 0.5% chloramine-T solution. The tooth crowns were removed with the use of a diamond disk (SP 1600 Microtome, Leica, Nu Block, Germany) at the level of cementodentinal junction. The teeth were mounted in acrylic molds so that a 3-mm area at furcation area was left out of the acrylic resin to provide a space for placement of a colloidal silver gelatin sponge (Geltamp, Roeko-Coltène/Whaledent, Langenau, Germany). The latter would be used after soaking in saline and act as a matrix to pack materials for the repair of perforated furcation area.

A #1/2 round bur, was placed perpendicular to the furcation floor and parallel to the long axis of the tooth, to create perforations. Then sizes 1 to 4 of Peeso Reamers (Dentsply Maillefer, Ballaigues, Switzerland) were used to enlarge the perforation as wide as 1.3 mm. The height of the walls of the perforated area were measured with a periodontal probe to standardize them at 2 mm. Samples with the height of dentinal wall less than 2 mm, were excluded from the study. If the thickness of the dentin was over 2 mm, the extra dentin was removed with a disk. All samples were rinsed with normal saline to eliminate the resultant debris.

The samples were assigned to 4 groups (*n*=20) based on the material used for perforation repair and use of CH. In group 1, white ProRoot MTA (Dentsply, Tulsa Dental, Tulsa, OK, USA) was used according to the manufacturer’s instructions with the powder to liquid ratio of 3:1. A special MTA carrier was used to place material in the perforation site. After removal of excess WMTA from the pulp chamber with a wet cotton pellet, a cotton roll impregnated with phosphate-buffered saline (PBS, Dulbecco's Formula Modified, ICN Biochemicals, England) was placed on the repair material with another being placed under the furcation area. Then the samples were wrapped in wet gauze pieces and incubated in closed containers at 37^º^C under 100% relative humidity for 7 days.

In group 2, CH powder (Golchay, Tehran, Iran) was mixed with distilled water to achieve a hard consistency. The paste was transferred to the pulp chamber cavity with the use of an amalgam carrier and packed with a plugger. After 7 days of incubation at 37^º^C and 95-100% humidity, CH was removed from the perforation area by irrigation with 0.5% NaOCl. Then, WMTA placement followed the same procedures as described for group 1. The samples were incubated in similar conditions. 

The procedural steps in groups 3 and 4 were similar to those in groups 1 and 2, except for the fact that CEM cement (BioniqueDent, Tehran, Iran) was used to repair the perforation in these groups and CH was placed in group 4 samples in the similar way. According to the instructions provided by the manufacture, the liquid was gradually added to powder to achieve the thick creamy mixture.

***Push-out test***

The push-out test was carried out using a universal testing machine (Model H5K-S; Hounsfield Test Equipment, Surrey, England). The restorative materials in the perforated area were subjected to a force at a crosshead speed of 0.5 mm/min in apical direction parallel to the tooth long axis by using a cylindrical bar measuring 1.1 mm in diameter, until dislodgment occurred. The maximum force before dislodgment was recorded in Newtons (N). The push-out bond strength was calculated in MPa using the following formula: bond strength (MPa)=force necessary for dislodgment (N)/bonded surface area (mm^2^) where bonded surface area was calculated as the *d×π×h *where *d *is the diameter of the perforated area and *h *represents height of perforation site*.*

***Statistical analyses***

After calculation of the means and standard deviations of bond strength values, two-way ANOVA was used to evaluate the effect of the CH on the push-out bond strength of the materials. Statistical significance was set at 0.05. SPSS software (SPSS version 18.0, SPSS, Chicago, IL, USA) was used for data analysis.

## Results

The mean±SD of the bond strength values are listed in Table 1. There were significant differences in resistance to dislodgement between the WMTA/CH and other groups (*P*<0.05). However, there were no significant differences in the bond strength values between other groups (*P*>0.05). 

## Discussion

The aim of the present study was to evaluate the effect of CH on the push-out bond strength of two endodontic materials (WMTA and CEM cement) used for perforation repair. The results showed the positive and significant effect of CH on the push-out bond strength of WMTA. However, for CEM cement the presence of CH before placement of the material in the perforation area did not affect the bond strength. 

In fact the bond strength of perforation repair materials is indicative of their resistance to dislodgment by occlusal forces and forces resulting from placement of restorative materials, such as amalgam, over the them [2, 8, 13]. In the present study, push-out bond strength was used to evaluate the resistance to dislodgment, which is a reliable technique based on previous studies [13]. The force required to condense amalgam varies from 5.5 to 9.17 MPa, depending on the size of the condenser [10, 21]. The materials evaluated in the present study have rather favorable bond strength in this context; therefore, WMTA and CEM are used as perforation repair materials due to their favorable properties [3, 4, 26, 27]. 

In addition to the materials used for perforation repair, some other factors should be considered in the management of this challenge, including the presence of inflammation in the area and the resultant acidic environment [10, 28]. On the other hand, due to their inflammatory nature, the physical properties of the perforation repair materials, such as WMTA, are influenced negatively [10].

**Table 1:** The mean (SD) of bond strength in different groups

**Repair material **	**Bond strength (MPa)**
**WMTA+CH**	13.08 (1.8)
**WMTA**	8.79 (2.4)
**CEM+CH**	9.06 (1.98)
**CEM**	8.03 (0.98)

CH has always attracted attention as an antimicrobial agent. The antimicrobial properties of its paste are attributed to the ionization to Ca^+^ and OH^-^ and creation of an alkaline environment [24, 29], making its application in the perforation area justifiable because there is the risk of microbial contamination, especially in old perforations [30]. 

Some studies have shown that CH results in degeneration or demineralization of root dentin and has a negative effect on the mechanical properties of dentin due to the alkaline pH of CH denaturing the organic proteins of dentin such as collagen [12].

However, some other studies have refuted such a hypothesis [19]. In the present study, such negative effects were not observed. 

The bond strength is influenced by different factors, including moisture and exposure to PBS due to the presence of phosphate ions in the area [20, 31, 32]. The materials used in the present study rely on moisture for setting. In the present study, after placement of the materials in the perforated area, a PBS-impregnated cotton pellet was placed under the furcation area to simulate the tissue fluid conditions and make phosphate ions available during the setting process. WMTA is mainly composed of Portland cement and its setting mechanism involves hydration of powder particles during which CH reacts with the phosphate of the interstitial fluid and produces a layer of hydroxyapatite [32]. Synthesis and maturation of hydroxyapatite crystals fills the microscopic spaces between WMTA and dentinal walls. In addition, tag-like structures from this intermediary layer penetrate into the dentinal tubules and precipitate along the collagen fibrils, improving the mechanical retention [8, 21, 33]. CEM cement also consists of different compositions of calcium and the difference between its setting reaction and that of WMTA lies in the fact that it can form a layer of hydroxyapatite on its surface in an endogenous manner because it contains phosphate compositions in contrast to WMTA [34-37]. CEM has the indigenous source of phosphate, which gives it the capability of producing HA crystals even in the presence of distilled water. WMTA lacks this source of phosphate and is dependent on external sources (*i.e. *tissue fluids) for its bioactivity. Therefore, adding PBS to the experiment makes a significant change for WMTA but has no effect on CEM cement.

Based on the results of a study by Weng *et al.* [8], synthesis and maturation of hydroxyapatite crystals depends on the concentration of the available ions. The availability of residual calcium ions from CH in the perforation site and their reaction with the phosphate ions in the area probably increase the bond strength of the materials evaluated with concomitant use of CH, which was higher with the use of WMTA compared to CEM cement. 

Under the limitations of the present study, it can be concluded that application of CH for one week before placement of WMTA yields favorable results. However, application of CH did not have an adverse effect on the bond strength of CEM cement. Nonetheless, based on the results of previous studies, the effect of CH on the microorganisms in the perforation area cannot be ignored; so it is recommended to use CH in perforation sites (especially old perforations) before placement of the repair materials.

## Conclusion

Calcium hydroxide elevates the push-out bond strength of WMTA used for repair of furcation perforation. However, it did affect the bond strength of CEM cement. Due to possible infection of the old perforations, if perforation repair with MTA is intended, placement of CH can provide the advantage of proper disinfection besides increasing the bond strength of repair material.
